# Effect of microbial fuel cell operation time on the disinfection efficacy of electrochemically synthesised catholyte from urine

**DOI:** 10.1016/j.procbio.2020.10.014

**Published:** 2021-02

**Authors:** I. Merino-Jimenez, O. Obata, G. Pasternak, I. Gajda, J. Greenman, I. Ieropoulos

**Affiliations:** a Bristol BioEnergy Centre, Bristol Robotics Laboratory, University of the West of England, BS16 1QY, UK; b Biological, Biomedical and Analytical Sciences, University of the West of England, BS16 1QY, UK; c Laboratory of Microbial Electrochemical Systems, Faculty of Chemistry, Wroclaw University of Science and Technology, 50-370, Wroclaw, Poland; d Instituto de Microelectrónica de Barcelona, IMB-CNM (CSIC), Spanish National Research Council, C/ del Til·lers, Campus Universitat Autònoma de Barcelona (UAB), 08193, Bellaterra, Barcelona, Spain

**Keywords:** Microbial Fuel Cell (MFC), Ceramic membrane, Catholyte production, Electroosmotic drag, Urine treatment, Energy from waste, Urine

## Abstract

Microbial fuel cells (MFCs) offer an excellent solution to tackle some of the major challenges currently faced by humankind: sustainable energy sources, waste management and water stress. Besides treating wastewater and producing useful electricity from urine, ceramic MFCs can also generate biocidal catholyte *in-situ*. It has been proved that the electricity generation from the MFCs has a high impact in the catholyte composition. Therefore, the catholyte composition constantly changes while electricity is generated. However, these changes in catholyte composition with time has not yet been studied and that could highly contribute to the disinfection efficacy. In this work, the evolution of the catholyte generation and composition with the MFC operation time has been chemically and microbiologically evaluated, during 42 days. The results show an increase in pH and conductivity with the operation time, reaching pH 11.5. Flow cytometry and luminometer analyses of bioluminescent pathogenic *E. coli* exposed to the synthesised catholyte revealed killing properties against bacterial cells. A bio-electrochemical system, capable of electricity generation and simultaneous production of bactericidal catholyte from human urine is presented. The possibility to electrochemically generate *in-situ* a bacterial killing agent from urine, offers a great opportunity for water reuse and resource recovery for practical implementations.

## Highlights

• The MFC with the thickest ceramic membrane produced the best quality catholyte.
• MFC operation time contributes to the catholyte quality and killing properties.
• Catholyte from ceramic MFC (10 mm) reached pH 11 at day 42 and eradicated *E. coli*.

## Graphical abstract

**Figure:**
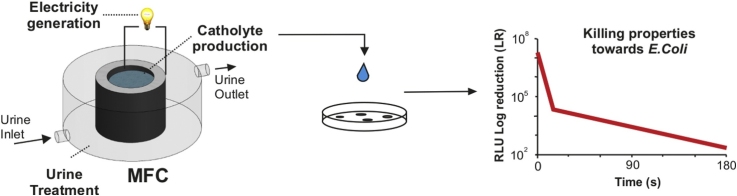

## 1 Introduction

The access to adequate sanitation facilities, while waterborne diseases threaten the human health, remain one of the major issues in the developing countries [1]. The development of a technology that can be domestically integrated, would offer the solution to the high cost and the infrastructure required by a centralized wastewater treatment plant [2]. In addition, an integrated sanitation technology that offers the possibility to generate electricity and recover nutrients from wastewater and urine would provide an added value [3,4]. In order to successfully promote nutrient reuse, an appropriate self-sustainable cost affordable system to decontaminate urine needs to be optimised.

Current disinfection methods are expensive due to high-energy consumption and non-sustainable due to excessive use of chemical agents. The electrolytic treatment, as well as disinfection, can be performed in wastewater electrolysis cell (WEC) *in situ*, where the externally applied electrical potential generates a variety of oxidants, such as reactive oxygen species (ROS) or reactive chlorine species [5,6]. Electrochemical systems (EC) have been proposed as a technology to successfully produce a disinfecting agent from various wastewater effluents [5,[7], [8], [9]]. These systems provide the *in-situ* generation of killing agents without the addition of chemical compounds, capable of disinfecting a wide range of pathogens. However, energy consumption is required in the EC disinfection systems, which needs to be provided by an external source, *i.e.* solar energy [5,10]. Human urine could serve as an example of an ideal electrolyte for electrochemical systems, being a source of nutrients [11,12], hydrogen [6] and electrical energy with the use of Microbial Fuel Cell (MFC) technology [[13], [14], [15], [16]], with simultaneous extraction of nutrients in the form of struvite [17,18], ammonia [19], and other by-products, such as the catholyte that can potentially be used as a disinfectant [20].

**Fig. 1:**
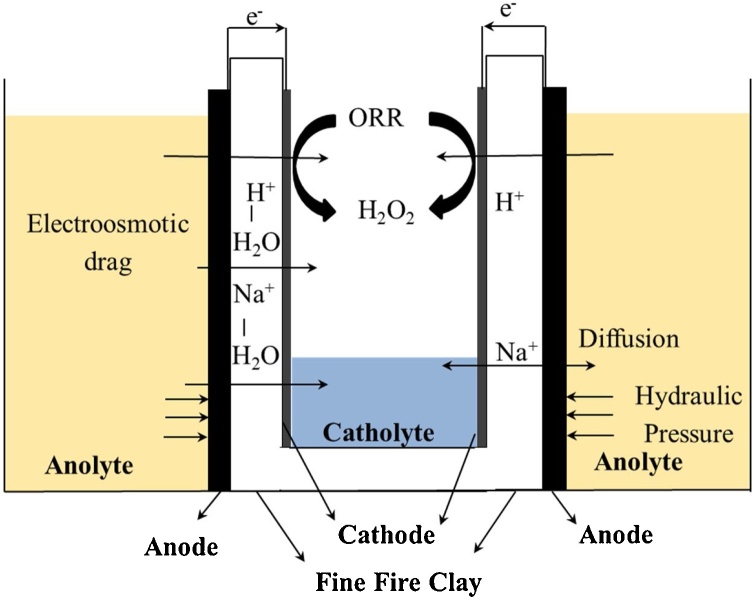
Schematic of the side view of a cylindrical ceramic microbial fuel cell and the effect of all the parameters that contribute to the catholyte generation.

In the MFCs treating urine, anodic bio-electrochemical reactions oxidise the organic matter from the urine releasing electrons, which are captured by the anode and transferred to the cathode *via* the external circuit, resulting in the generation of electricity. While the anodic MFC environment has been proven to have killing properties against pathogenic *Salmonella enteritidis* [21], as well as *Hepatitis* B antigens [22], ceramic MFCs have been reported to produce bactericidal catholyte in the cathode chamber [20,23]. Fig. 1 shows a schematic diagram of the parameters that contribute to the catholyte generation: i) oxygen reduction reaction (ORR) taking place at the cathode, ii) electro-osmotic drag of water molecules and ion transfer from anode to cathode chamber, iii) diffusion due to a concentration gradient on both sides of the ceramic membrane and iv) hydraulic pressure affected by porosity of the material and the MFC design [20,24]. While the main characteristics of the antimicrobial catholyte produced within the MFC are attributed mainly to high pH as a result of power generation [20,23], other parameters might influence catholyte quality.

Although the understanding of the electrochemical synthesis of catholyte from ceramic MFCs has been improved [17,20,[23], [24], [25], [26]], the full composition and properties of the catholyte remains unaddressed. Moreover, the effect of the different parameters that contribute to the catholyte generation, its quality, composition and applicability needs to be individually evaluated to obtain the best catholyte quality. These parameters include the type of anolyte used as feedstock, its organic and inorganic content, ionic strength and pH (*i.e.* wastewater, urine, industrial wastewater), the nature of the ceramic membrane (composition, porosity, thickness) [24,27], the MFC power output [28,29] and operation time (*i.e.* extraction of catholyte after one day, one week or one month of operation). A correlation between power generation from the MFCs and the ceramic thickness has been reported, with the most powerful MFC having the thinnest membrane. However, the best quality of catholyte with the highest pH was obtained from the thickest membrane MFC [24]. A study on the effect of the physico-chemical properties of the fine fire clay on the MFC power generation and catholyte quality revealed an increase in MFC power output and catholyte quality with the open porosity of the ceramic, obtaining the best performance with ceramic membranes of an open porosity of 16 % [26].

For as long as the MFC is generating electricity, a constant ion transfer from the anolyte to the cathode chamber takes place and electrochemical reactions taking place at the cathode, generating species that contribute to the catholyte composition. Therefore, there is a time element that is highly affecting the concentration of species residing in the cathodic chamber, and therefore the catholyte quality and killing properties.

In this work, the changes in the catholyte composition with the MFC operation time was analysed. Microbial analysis was also performed using plate count method and flow cytometry (FCM) for an accurate determination of live, dead, and total bacteria, and to determine whether the viable count decreased as the catholyte composition changes and becomes highly alkaline. The number of viable bacteria in the catholyte samples was also estimated. This study also allows a comparison of the catholyte killing properties with the thickness of the ceramic membrane.

## 2 Materials and methods

### 2.1 MFC construction

**Fig. 2:**
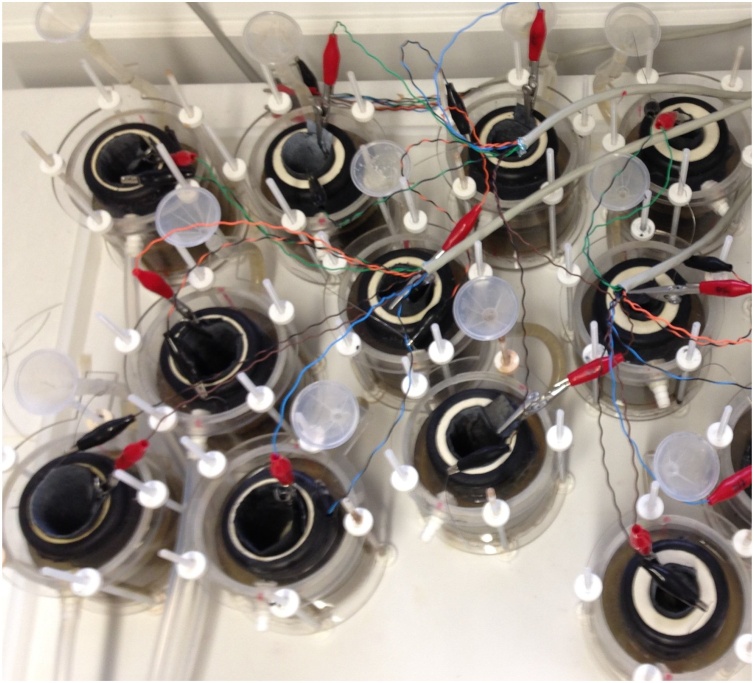
Picture of the top view array of microbial fuel cells with ceramic membranes of three different thicknesses.

A total of 12 MFCs were assembled using fine fire clay (FFC) cylinders as the membrane with three different thicknesses, 2.5, 5 and 10 mm, as previously described [24]. The chemical composition typical for fire clays includes 23–34 % Al_2_O_3_, 50–60 % SiO_2_ and 6–27 % loss on ignition together with various amounts of Fe_2_O_3_, CaO, MgO, K_2_O, Na_2_O and TiO_2_. The specific composition of the fine fire clay used herein cannot be disclosed. The open porosity of the ceramics used was 11.7 % and the ionic conductivity was 0.01 S cm^−1^. A picture of the array of MFCs is shown in Fig. 2. Carbon veil (30 g/m^2^, PRF Composites, Dorset, UK) was used as the anode electrode with a total surface area of 90 × 27 cm^2^ which was folded and wrapped around the ceramic cylinder. The cathode electrodes, with a surface area of 65 cm^2^, were prepared by spreading a mixture of activated carbon (GBaldwin&Co, 80 g · 140 m L^−1^ solution), polytetrafluoroethylene (PTFE) 60 % wt. (Sigma-Aldrich) and distilled water, on one side of the carbon fibre veil treated with 30 % PTFE to achieve hydrophobicity (30 g m^−2^, PRF Composites, Dorset, UK) [30]. The cathodes were introduced by attaching the activated carbon layer to the walls in the ceramic cylinders, completing the MFC assembly. A carbon-based cathode electrode was chosen since, besides following the peroxide pathway, it offers high conductivity, high durability, high mechanical strength, and high surface area at an affordable cost [31]. The MFC was then embedded in an acrylic tubular container which occupies approximately 200 mL of urine. As current collectors, a stainless steel wire (0.5 mm, Scientific Wire Company) was threaded through the anode electrodes and a stainless steel crocodile clip was connected to the cathode electrodes.

### 2.2 MFC operation

The inoculation process was performed during three days using a mixture of 50 % activated sewage sludge supplied from the Wessex Water Scientific Laboratory (Saltford, UK) and 50 % fresh urine, collected from healthy individuals. The MFCs were under open circuit for the first two hours of inoculation, after which a 2 kΩ external resistance was connected. Relatively high resistance was used in order to avoid high variance in performance of the established communities as explained by Pasternak et al. [32]. After the inoculation phase, the feedstock was 100 % fresh undiluted urine in a continuous upward flow system (9 mL h^−1^ giving a hydraulic retention time (HRT) of 22 h) using a 16-channel peristaltic pump (205 U, Watson Marlow, Falmouth, UK). Once the MFCs were matured, the external resistance connected to each MFC was changed to 80 Ω to achieve optimum performance, which remained constant throughout the experiment. This resistor was selected from the polarisation experiment (Fig. S1), as the optimum resistor to achieve maximum power performance. The open circuit voltage of the MFCs observed before the polarisation started was 598 mV ± 5 mV, with no significant difference observed due to the ceramic thickness. All experiments being performed at room temperature 22 ± 2 °C.

The current and power generated was calculated from value of the external resistance connected to each MFC and the individually monitored (KEYSIGHT, 34972A LXI data acquisition/Switch unit) cell voltage against time [15]. The cathode potentials were constantly monitored *versus* the Ag/AgCl reference electrode, by introducing the reference electrode in the cathode compartment. The anode potential was sporadically measured by temporarily stopping the feeding line and introducing the Ag/AgCl reference electrode into the inlet tube in contact with the anolyte. During operation, the cathode redox potential was also monitored and the anode potential was calculated using Eq. (1):

(1) $$E_{cell}=\left(E_{Cathode}-E_{Anode}\right)-\sum IR$$

Where ∑IR includes combination of losses from the membrane internal resistance and the electrical circuit.

### 2.3 **E**lectrochemcial impedance spectroscopy (EIS)

In order to further study the membrane resistance, electrochemical impedance measurements were done by using a μAutoLab Type III with a frequency response analyser FRA 2. A frequency interval from 100 kHz to 10 mHz was employed for the measurements at 10 mV AC amplitude. The tests were run in the operating MFCs, for the anode and cathode electrodes *versus* reference electrode (Ag/AgCl), where the reference electrode was placed in the anode and cathode chamber, respectively. The EIS was also run for the complete MFC, where the ohmic resistance of the ceramic membrane is also included. The electrical resistance of the membrane could be identified from the Nyquist plots.

### 2.4 Chemical and microbiological analysis

Once the MFCs reached steady state, the existing catholyte was completely drained from each MFC before the experiment started (time 0), in order to start quantifying and characterising the catholyte. The amount of catholyte accumulated in each cylinder, its pH and conductivity were measured almost on a daily basis. A total of 4 mL of catholyte sample was collected from each FFC MFC for analysis periodically every 7 days of operation. A Hanna 8424 pH meter was used to measure the pH and the conductivity was measured using a 470 Jenway conductivity meter (Camlab, UK). To determine the concentration of anions and cations in the urine and in the catholyte samples, an ion chromatograph 930 Compact IC Flex (Metrohm, UK) was used. The potassium dichromate oxidation method (COD HR test vials, Camlab, UK) with an MD 200 photometer (Lovibond, UK) was used to analyse the chemical oxygen demand (COD).

Analysis of disinfecting properties of the catholyte was conducted with the use of flow cytometry. All samples were centrifuged and washed with using 0.2 μm-filtered NaCl solution (0.85 %). Resuspended samples were stained with BacLight™ Green Bacterial Stain according to the manufacturer’s instructions (Life technologies, USA).

The measurements were carried out using BD Accuri™ C6 flow cytometer (BD, USA). The samples were analysed using side scatter (SSC), forward scatter (FSC) and green fluorescence (FL1) signals. Combination of FSC and SSC signals were used for discriminating signal derived from bacterial cells and the noise. Filtered NaCl solution was used as the control sample. Gating for the combined FL1 and FSC signals was set up in order to quantify the living, dead and injured cells. Bacterial killing analysis was conducted by exposing bioluminescent *Escherichia coli* to catholyte obtained at various time points from MFCs of different membrane thickness. Collected catholyte was tested as a biocidal agent on bioluminescent *Escherichia coli* Nissle 1917 pGLITE. The bacteria were grown on nutrient agar plates supplemented with appropriate antibiotics kanamycin (10 μg/mL) to maintain the lux plasmid, transferred to nutrient broth and incubated at 37 °C overnight. Subsequently, 0.5 mL of bacterial culture was added to a clean 1.5 mL Eppendorf tube (Fisher Scientific, Loughborough, United Kingdom) for bioluminescence measurement using a single-tube FB12 luminometer (Berthold Detection Systems, Germany) to quantify relative light units (RLU) in 0.5 mL of phosphate buffer saline (PBS pH 9.3) as the control and to the same amount of catholyte to evaluate the biocidal efficacy. PBS was adjusted to pH 9.3 using NaOH, to discount the impact of the high pH on the killing efficacy. The automated protocol included a 3 s delay to allow the reading of measurements. Bacterial bioluminescence was recorded every 15 s for the first minute and then at 60 s intervals. Units of relative light emission (RLU) were transformed to log^10^ values and plotted to show kill kinetics of the target organism.

## 3 Results and discussion

### 3.1 MFC operation

**Fig. 3:**
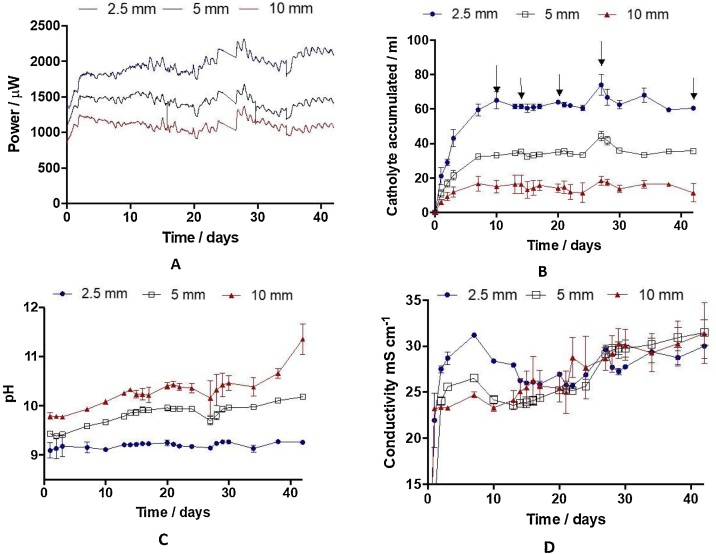
Timeline of the different parameters monitored for the microbial fuel cells with ceramic membranes of 2.5, 5 and 10 mm thick: (A) Power, (B) catholyte volume accumulated, (C) catholyte pH, (D) catholyte conductivity.

Fig. 3A shows the average power performance of the MFCs *versus* time, while Fig. 3B–D illustrate the amount of catholyte collected, its pH and conductivity, respectively, for the MFCs with three different thicknesses 2.5, 5 and 10 mm. Power output clearly correlates with membrane thickness and this is due to the membrane resistance [26,33]. This evaluation looks into the several physico-chemical properties of newly formed catholyte with respect to MFC operation time and its power performance. The maximum absolute power was generated by the MFC with the thinnest membrane, 2.1 mW, which is higher than previously reported values, 1.1 mW, for ceramic MFCs of similar size and treating the same urine volume [34]. COD analysis, performed at day 1, revealed a reduction of 15.5, 14.5 and 13 %, respectively for the thin, medium and thick ceramic MFCs, with an initial COD in urine of 8.1 ± 0.4 g L^−1^. Before the experiment started, the catholyte generated had been completely drained causing a slight decrease in power output as observed in Fig. 3A. After that, the power generated constantly increased for the first two days, while the amount of catholyte generated also increased rapidly during the first 7 days to remain constant at 15, 35 and 60 mL respectively, for the MFCs with the thickest, medium and thinnest membranes (Fig. 3B).

The increase of the catholyte accumulated with the ceramic thickness is due to the predominant hydraulic pressure and free osmotic movement in the thinner ceramics. High water flux usually leads to high reverse salt diffusion [35] that is the backward transport of salt ions across the membrane. Current generation however can effectively prevent the reverse salt diffusion, as recently reported in Osmotic MFCs [36] therefore to successfully operate ion separation it is required to optimise the power generation, as well as the membrane thickness.

As shown in Fig. 3C, the pH of the catholyte from the MFC with the thinnest membrane barely increased during the experiment, varying from 9.1 to 9.3, while the pH of the catholyte generated from the MFCs with the 5 and 10 mm thick ceramic membrane showed a more abrupt increase, from 9.4 and 9.8, respectively on day 0, to 10.3 and 11.3, on day 42. This might be due to the effect of the accumulation of OH^−^ in the catholyte, which rapidly increased the pH of the catholyte in the MFCs with the thicker membranes. As shown in Fig. 3D after 20 days of operation, the conductivity of the catholyte collected from all MFCs was similar, reaching 30 mS cm^−1^. Those parameters could be compared with that of the urine used to feed the MFCs: pH = 9.2 ± 0.5, conductivity = 29.1 ± 0.4 mS cm^−1^. The pH and conductivity of the catholyte collected from the MFC with the thinnest ceramic separator remained at constant levels for the duration of the experiment. These values were similar to those of the anolyte, suggesting the osmotic force is dominating the flux through the thin ceramic. Therefore, the ceramic thickness plays an important role in catholyte accumulation in this set up and for the purpose of the catholyte collection, it would be paramount to take this parameter into account. The results also show that the pH and ion separation from the catholyte from the MFCs with the medium and the thickest ceramic membranes, show gradual increase in time due to the lower osmotic influenced electroosmotic flux. Therefore, the MFC operation time is a parameter that worth analysing, since the catholyte quality and capability to kill pathogens may be time dependent.

Table 1 shows the average anode and cathode potential, the catholyte pH and the MFC voltage measured for each MFC type on day 42. Table 1 also shows the losses due to the membrane, electrolytes and electrodes resistances, calculated using Eq. (1). The average loss (∑IR) was 4.6 mV, 86.3 and 206.5 mV for the MFCs with the thinnest, medium and thickest membranes, respectively. As it was expected, the thicker membranes lead to higher ohmic losses [37]. The anode electrode potential was similar for all the MFCs with an error of ±10 mV, suggesting a similar performance from the anode biofilm. On the contrary, the cathode electrode potential showed considerable differences depending on the membrane thickness, which can be attributed to the differences in the catholyte pH. It is generally accepted that the ORR on activated carbon takes place through a 2 e^−^ pathway, according to the following reaction [37]:

(2) $$O_{2}+H_{2}O+2e^{-}\to O_{2}H^{-}+{OH}^{-}$$

**Table 1:** Comparison of the anodic and cathodic potentials, catholyte pH, microbial fuel cell (MFC) voltage and average loss of the ceramic MFCs with three different thicknesses operating under optimum external resistance at operation day 42.

Membrane Thickness	Average anodic potential *vs* Ag/AgCl / mV	Average cathodic potential *vs* Ag/AgCl / mV *E(O_2_/HO_2_^−^)*	Catholyte pH	MFC voltage measured / mV	Average loss (∑IR) calculated from equation (1) / mV
2.5 mm	−414.5	2.5	9.27	412.35	4.6
5 mm	−407.5	25.8	10.10	346.98	86.3
10 mm	−427	86	10.72	306.5	206.5

However, there is a substantial disagreement on the reaction mechanism, although most of the authors establish that the superoxide ion $O_{2}^{∙-}$ and the intermediate hydroperoxyl radical $\;{HO}_{2}^{∙}$ are involved [39]. The cathode potentials in Table 1 decreased per pH unit according to Nernst Eq. (3), upon changing the pH to more alkaline values [38].

(3) EO2HO2- =Eo'-2.3RTnF∙logO2H-∙[OH-]PO2∙PH2O

Where *E(O_2_/HO_2_^−^)* is the cathode reduction potential, *E^o’^* is the standard potential of the reduction reaction (−0.076 mV *vs* SHE), *R* is the gas constant, *T* is the temperature, *n* is the number of electrons transferred, *F* is the Faradays’ constant, *P_O2_* and *P_H2O_* are the pressure of the *O_2_* and *H_2_O* (1 atm). For this work, it is assumed that oxygen is the only oxidant reduced at the cathode. The concentration of hydroxyl ions formed can be calculated from the increase in the pH and therefore the theoretical concentration of peroxide ions formed during the ORR according to reaction (2) could be calculated from Eq. (3). The concentration of OH- formed during the MFC operation was calculated from the pH difference between the catholyte collected under load and that under open circuit conditions, assuming that the pH difference is only due to the OH- formation in reaction (2). At day 42, the concentration of O_2_H- in the catholyte was 1.3, 1.42 and 1.7 mol m^−3^ (0.03, 0.033 and 0.04 mL L^−1^) for the MFCs with the ceramic membrane of 2.5, 5 and 10 mm, respectively.

**Fig. 4:**
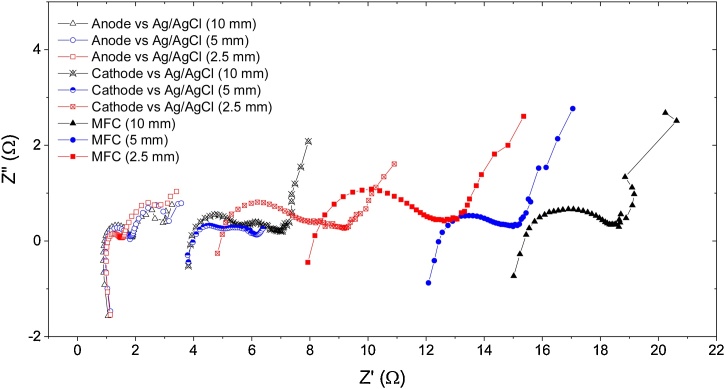
Nyquist plots of the anode *vs.* Ag/AgCl, cathode *vs.* Ag/AgCl and MFC impedance of the three types of MFCs with ceramic thickness of 2.5, 5 and 10 mm.

Fig. 4 shows the Nyquist plots of the anode *vs.* Ag/AgCl, cathode *vs.* Ag/AgCl and MFC impedance for the three type of MFCs used in this study (2.5, 5 and 10 mm ceramic membranes). As shown in the graph, the anode polarization resistance is similar in all the MFC types, having a fairly low contribution to the total MFC internal resistance. On the contrary, the cathode polarization resistance shows differences in the cathodic resistance, with 5 ohms for the cathode in the MFC with the thinnest membrane (2.5 mm), and 4 ohms for the cathode in the MFCs with the medium and thick membranes. The difference in the cathode polarization resistances among the MFCs is due to the changes in the catholyte pH, 10.5, 10.4 and 9.77 for the thick, medium and thin MFCs, respectively; since the pH directly affects the ORR kinetics. A clear decrease in the MFC ohmic resistance with the ceramic thickness can be observed. Given the anode polarisation, the cathode polarisation and the MFC resistance, the ohmic resistance of the ceramic membranes can be extracted, obtaining 2.1, 7.4 and 10.4 ohms for the thin, medium and thick ceramics, respectively.

### 3.2 Catholyte quality

**Fig. 5:**
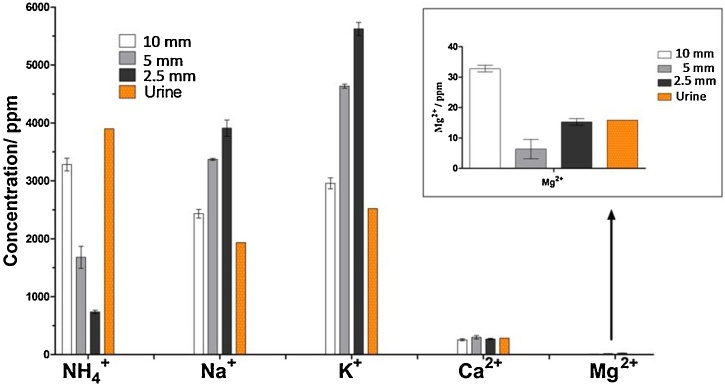
Concentration of cations in urine from the inlet and in the catholyte collected on day 10 from ceramic microbial fuel cells with three membrane thicknesses: 2.5 mm, 5 mm and 10 mm, measured by ion chromatography.

Fig. 5 shows the concentration of cations in the urine (last column) and in the catholyte collected from the ceramic MFCs with different thickness, on day 10. An increase on the concentration Na^+^ and K^+^ with the thickness of the ceramic membrane can be observed, being the concentration on the catholyte collected from the thinnest ceramic MFC similar to the concentration of urine and becoming more concentrated for the MFCs with thicker membranes. Taking into consideration the amount of accumulated catholyte on the day 10 (Fig. 3B), the total amount of transported cations such as Na^+^ and K^+^ was the highest in the MFC with the highest power output. However, the effect of the electro-osmotic drag phenomenon becomes more apparent when the medium and thick membranes are used because of a lower contribution of effects due to passive osmosis through the membrane and hence why the increase in the concentration of the positive ions with the ceramic thickness [24]. A much lower concentration of Ca^2+^ and Mg^2+^ was observed in the catholyte, which is understandable considering initial concentration of these cations in urine. This results suggest that monovalent ions such as Na^+^, K^+^ and potentially NH_4_^+^ are electrically driven from the anode chamber, as the NH_4_^+^ has been reported as the proton shuttle in previous studies [39]. On the contrary, the concentration of NH_4_^+^ decreased with the thickness of the membrane. That is related with the changes in the pH of the catholyte from the different MFCs (Fig. 3C). Ammonia in gaseous state (NH_3_) and aqueous ammonium ion (NH_4_^+^) are in equilibrium at a pH of approximately 9, with higher pH's forcing more NH_4_^+^ into the gas form, being both concentrations directly related with the pH by the following equation:

(4) $${NH}_{4}^{+}↔{NH}_{3}+H^{+}$$

$$\frac{\left[{NH}_{3}\right][H^{+}]}{\left[{NH}_{4}^{+}\right]}={5.7x10}^{-10}$$

That explains the decrease in the concentration of NH_4_^+^ with the thickness of the ceramic membrane, since the thicker the ceramic membrane, the higher the pH of the catholyte in it and the higher the concentration of NH_3_ in gas form [40]. The NH_3_ stripped into the air could be collected from the catholyte chamber for nitrogen recovery. The selectivity of the membrane for the positive ions becomes more noticeable when the membrane is thicker. The NH_3_ production might also contribute to the disinfection mechanism [20] as in Fig. 5, ammonium concentration in the catholyte of highest pH collected from the thickest membrane shows lowest concentration of ammonium. This allows to assume that the largest fraction of the NH_4_^+^ was already transformed to the NH_3_ in the catholyte of highest pH, as the inactivation is mainly attributed to the molecular form (NH_3_) rather than the ionized form (NH_4_^+^) [41].

**Fig. 6:**
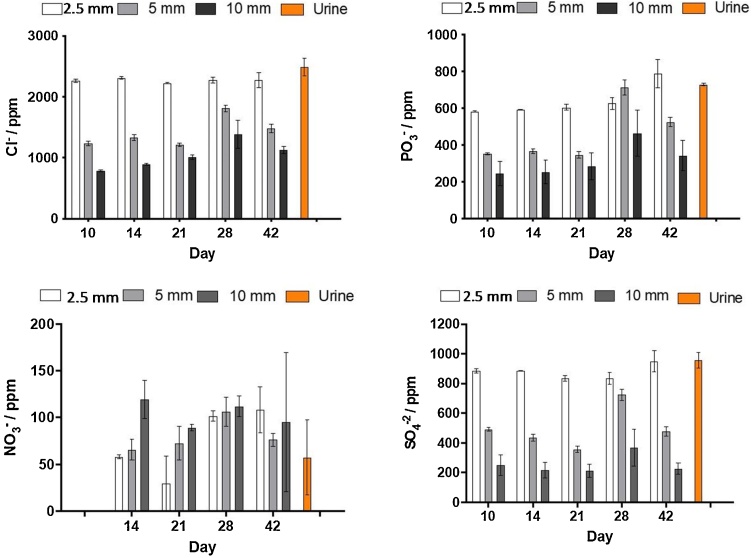
Concentration of anions in the urine from the inlet and in the catholyte collected at different operation times (10, 14, 21, 28, 42 days) from ceramic microbial fuel cells with three membrane thicknesses: 2.5 mm, 5 mm and 10 mm.

Fig. 6 shows the concentration anions in the catholyte collected from the different types of MFCs after 10, 14, 21, 28 and 42 days of operation. As shown in the figure, a correlation between the ceramic thickness and the concentration of anions in the catholyte can be observed, decreasing the concentration of Cl^−^, PO_3_^−^ and SO_4_^−2^ with the ceramic thickness, whereas the concentration of nitrate increases. This is due to a higher overall effect of the charge compensation and the electro-osmotic drag in thicker ceramics [24]. The concentration of nitrite was also measured showing negligible values in all cases. An increase in the concentration of nitrate with time can also be observed, which indicates a constant transfer of nitrate molecules through the membrane or a product of a redox reaction involving nitrate ions taking place in the cathode chamber. However, the concentration of nitrate anions is over 100-fold lower than that of chloride and sulphate. No significant accumulation of Cl^−^, PO_3_^−^ and SO_4_^−2^ can be appreciated with time, suggesting an initial molecule transfer which is not time dependent. Chloride can be electrochemically transformed to reactive chlorine species such as hypochlorite ion (ClO^−^) at the pH higher than 8, the majority of the chlorine presented in the form of ClO– [42].

Cl‾ + 2OH‾→ ClO‾ + H2O + 2ē

As shown Fig. 6, the chloride concentration does diminish as the membrane thickness increases, this might support the pH related transformation into hypochlorite and along with high pH (-OH action) and -OCl oxidation [43] might be responsible for the disinfection efficacy that lead to amino acid degradation and hydrolysis [43].

A more significant decrease in the concentration of SO_4_^2−^ with the ceramic thickness can be observed in comparison with PO_3_- ions. This can be related with the molecule charge (valency), the hydration number (number of water molecules attached to each ion) and overall hydrated molecule size.

The Total Viable Count (TVC) analyses performed (Supporting Information, Fig. S2) demonstrates that the catholyte obtained from the thickest FFC MFCs showed up to 2-log reduction in microbial growth in comparison to the analysed anolyte in the same reactor. This is probably due to an increased pH and conductivity of the generated active basic solution, since the higher the catholyte pH, the lower TVC.

**Fig. 7:**
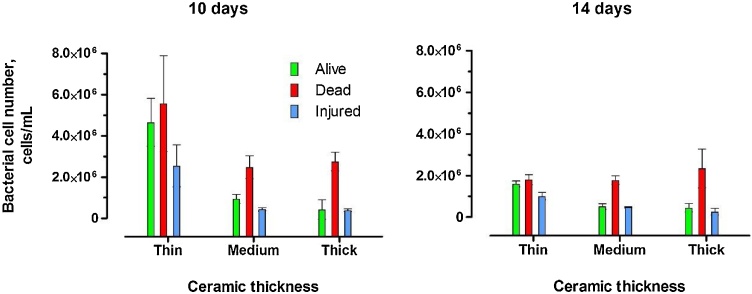
Bacterial cell number from the catholyte collected from the thin, medium and thick ceramic microbial fuel cells on days 10 and 14 of operation.

As revealed by flow cytometry measurements (Fig. 7), in the initial days of MFCs operation, the lowest number of living bacteria was reported for the thick membranes. A noticeable decreasing trend of living cells was observed in relation to the membrane thickness both after 10 and 14 days of operation. The overall bacterial community was smaller after 14 days of operation for all of the MFCs when compared to 10 days. Such a result may suggest the ongoing complete lysis of the cells, when suspected to the accumulating ionic species. Interestingly, the decreased survivability of the bacteria with increased thickness was correlated with the accumulation of cationic species (Na^+^ and K^+^). Such an outcome could therefore have emerged from the presence of hydroxide ions and a resulting, highly alkaline conditions which led to the lysis of bacterial cell walls.

**Fig. 8:**
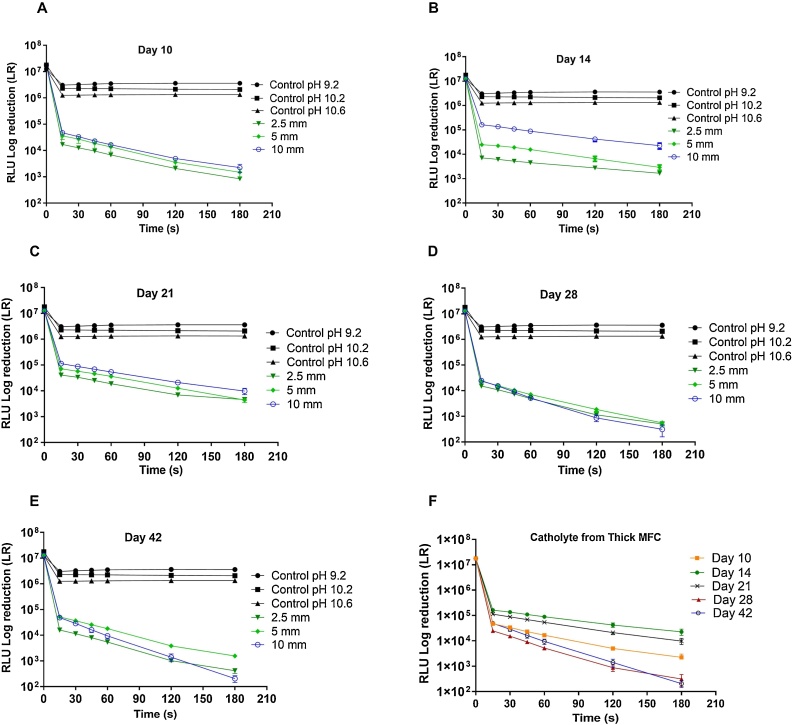
Log Reduction in bioluminescence (viability) of bioluminescent pathogenic *E. coli* exposed to catholyte collected from MFCs of different membrane thickness at day (A) 10 (B) 14 (C) 21 (D) 28 (E) 42. (F) Reduction in bioluminescence pathogenic *E. coli* exposed to catholyte collected from MFCs with the thickest membrane at different time points. Control is phosphate buffer saline (pH 9.2, 10.2 and 10.6 corresponding to the pH of the catholyte solutions).

Further analyses of the effect of the MFC operation time were performed by luminometer analyses (Fig. 8). Since bioluminescence has been shown to have a direct correlation with the bacterial viability [20], these results highlight the catholyte ability to kill pathogenic bacteria used in this study. As can be seen from Fig. 8, less than 1 log-fold reduction was recorded when the control solutions with pH up to 10.6 was added to the *E. coli*. All catholyte solutions gave significantly higher log-fold reduction that the corresponding controls of similar pHs. The addition of catholyte solutions collected at day 10 of the MFC operation already resulted in up to 4 log-fold reduction in *E. coli* viability within 180 s. The results show more than 1 log-fold reduction per minute from all catholyte solutions obtained from the thin, medium and thick membrane MFCs. Furthermore, *t*-test analysis showed that the differences in the killing efficacy of the catholyte and the control solutions of similar pHs were very significant. The kill curves recorded from catholyte solutions obtained in this study from urine samples are in agreement with those reported from catholyte generated from wastewater treated in MFCs [24].

Results of the luminometer analysis of catholyte collected on day 28 revealed much stronger solutions, resulting in more than 4 log-fold reduction in the *E. coli* viability (Fig. 8D). Similarly, catholyte solutions collected at day 42 brought about the highest level of pathogen killing. Catholyte from the medium ceramic MFCs resulted in almost 4 log-fold reduction while the catholyte from the thin and thick ceramic MFCs gave 4 and 5 log-fold reduction in pathogen levels, respectively. The catholyte generated from thick ceramic MFC at day 42 was significantly more efficacious than the catholyte from both thin and medium ceramic MFCs. In effect, the catholyte collected at day 42 from the thick ceramic completely eradicated the pathogens within 180 s (Fig. 8E).

Fig. 8F shows the killing curve obtained from the catholyte solutions collected from the thick ceramic MFC, giving an indication of the impact of timing on the efficacy of the catholyte solutions. The catholyte solution collected from the thick ceramic resulted in killing rates of 1.3, 1, 1.1, 1.6 and 1.7 log-fold reduction per minute for day 10, 14, 21, 28 and 42 respectively. *T*-test analysis showed that the killing rates recorded from catholyte from thick ceramics was significantly higher when collected at day 28 and 42 that at the other days. This is partially due to the fact that the MFCs with the thickest membrane also had the highest anode and cathode potentials, as shown in Fig. 3A and Table 1, which can lead to an increased bacterial killing. Moreover, the chemical analyses demonstrated the presence of higher concentrations of ions hydroperoxyl, nitrate, sodium and potassium as well as higher catholyte pH, which suggests the selective nature of the thick membranes. The results of this study are in agreement with previous research on the impact of membrane thickness on the selectivity and composition of catholyte [24].

Although the high pH of the catholyte is important in suppressing of the bacterial growth, another important factor affecting the microbial survival is the catholyte composition. The MFCs with the thickest membrane was also the one with the highest concentration of HO_2_^−^ in the catholyte, leading to an increased pathogen killing activity. High hydroperoxyl ion concentrations can result in the build-up of high concentrations of $∙O_{2}^{-}$ and $∙{OH}^{-}$ radicals, which may contribute to the bacterial killing properties of the catholyte by changing the oxidative-reductive potential (ORP) of the microenvironment, which in turn affects the bacterial growth and survivability [21,32]. Teh et al. [44], investigated the effect of hydrogen peroxide concentration on microbiological removal on greywater at H_2_O_2_ concentrations ranging from 0.25 to 2.5 mL L^−1^. The results showed that efficiencies were independent of the concentration of hydrogen peroxide at the range used, obtaining relatively high rates of bacteria inactivation, ranging from 96.01 % to 98.89 %. However, the exposure time to $∙O_{2}^{-}$ and $∙{OH}^{-}$ radicals can play an important role in the bacterial killing. A study conducted by Wagner et al. [45] showed that a contact time of 2 h and doses of up to 0.5 g L^−1^ (0.35 mL L^−1^) were needed to reach target bacteria concentration. The action of hydrogen peroxide has been reported to be rather slow, with bactericidal effects found even after 4 h for certain organisms. Thus, by extending the contact time between the pathogenic solution and the catholyte, the concentration of *E. coli* might reach even lower values. The reported studies are based on the exposure of the solution to only hydrogen peroxide.

For this work, it should also be considered that it is the combination of parameters such as pH, catholyte composition and anodic potential what contributed to the highest bacterial killing efficacy, recorded in the catholyte obtained from the thick membrane. Apart from the impact of current generation and ceramic thickness on the efficacy of the catholyte as a killing agent, there is an indication that time was also an important factor that determines the efficacy of the catholyte solution. It means that the longer the catholyte solution stays in the MFCs, the more efficacious it is as a killing agent. This is as a result of the accumulation of various cations and anions as well as the build-up of high concentrations of possible antimicrobial agents as NH_3,_ $∙O_{2}^{-}$ and $∙{OH}^{-}$ radicals in the catholyte solutions.

## 4 Conclusions

This work presents the study of the changes in the physico-chemical and microbiological properties of the catholyte generated by ceramic MFCs with the operation time. The composition of the catholyte constantly changed with time, especially for the catholyte formed in the MFCs with the thicker membranes. Overall, the result of this study showed that catholyte generated from closed circuit MFCs possesses strong bactericidal effects and pathogen killing properties towards *E. coli*. The highest killing rate overserved in this work was obtained from the catholyte generated from the MFCs with the thickest membrane (10 mm), as a result of a more efficient oxygen reduction reaction at higher pH and electro-osmotic drag across the thick membrane. The results also suggested that the best bacterial killing was obtained after 42 days of operation of the MFC, showing a correlation between the catholyte quality as a killing agent and the residence time. The optimization of parameters such as ceramic physico-chemical properties, ceramic thickness, MFC power generation and MFC operation time can highly contribute to the generation of more volume of a better quality catholyte. The generation of a pathogen-killing agent as a by-product of the urine treatment from MFCs, adds one more value to the urine treatment from MFCs, increasing the interest of such devices, especially for remote locations aiding sanitation and sustainable recycling. The use of cost affordable decentralized wastewater treatment systems offers an innovative sanitation technology for an efficient, safe and sustainable wastewater treatment.

## CRediT authorship contribution statement

**I. Merino-Jimenez:** Conceptualization, Data curation, Formal analysis, Investigation, Methodology, Writing - original draft, Writing - review & editing. **O. Obata:** Investigation, Methodology, Data curation, Formal analysis, Writing - review & editing. **G. Pasternak:** Investigation, Methodology, Data curation, Formal analysis, Writing - review & editing. **I. Gajda:** Investigation, Formal analysis, Methodology, Data curation, Writing - original draft, Writing - review & editing. **J. Greenman:** Conceptualization, Supervision, Writing - review & editing. **I. Ieropoulos:** Conceptualization, Funding acquisition, Supervision, Writing - review & editing.

## Declaration of Competing Interest

The authors declare that they have no known competing financial interests or personal relationships that could have appeared to influence the work reported in this paper.
