# Supplementary material for: Effect of microbial fuel cell operation time on the disinfection efficacy of electrochemically synthesised catholyte from urine
Source: Process Biochem. 2021 Feb;101:294–303. doi: 10.1016/j.procbio.2020.10.014 (PMC7893686; doi:10.1016/j.procbio.2020.10.014)
Supplement: Supplementary file 1 [file mmc1.docx]

**Supporting Information:**

Figure S1 Polarisation curves of the microbial fuel cells with different thicknesses (2.5, 5 and 10 mm), before the 42 days operation experiment started and with the cathode chamber empty of catholyte. Error bars indicate SEM with n = 4.

For the total viable count, the catholyte samples for the thick FFC MFCs were analysed in comparison with the control samples from the outlet of the equivalent reactor (FFC1, FFC2, FFC3) on the 14, 21 28 day of MFC operation The total viable count (TVC) of organisms was performed by a conventional serial dilution method with surface spreading of 0.1 mL of samples onto the petri dishes containing nutrient agar (Oxoid, UK), which were incubated aerobically at room temperature (22 °C) for 72 h. Microbial colony counts were expressed as log10 colony forming unit (CFU). Samples collected on the day 28 might show increased CFU numbers possibly due to the system flushing. A technical inconvenience led to an increased pumping of urine for 24 h on day 28.

Figure S2. CFU count obtained from the catholyte and anolyte samples collected on the 14, 21 and 28^th^ day of experiment.
